# Tailoring the Micropore Structure of 6FDA-Based Network Polyimide Membranes for Advanced Gas Separation by Decarboxylation

**DOI:** 10.3390/membranes13050461

**Published:** 2023-04-24

**Authors:** Yuxuan Zhao, Hongyan Wang, Xiangyun Liu, Xueping Zong, Jiangzhou Luo, Song Xue

**Affiliations:** Tianjin Key Laboratory of Organic Solar Cells and Photochemical Conversion, School of Chemistry & Chemical Engineering, Tianjin University of Technology, Tianjin 300384, China

**Keywords:** 6FDA-based network polyimide, micropore structure, gas separation, decarboxylation

## Abstract

The 6FDA-based network PI has attracted significant attention for gas separation. A facile strategy to tailor the micropore structure within the network PI membrane prepared by the in situ crosslinking method is extremely significant for achieving an advanced gas separation performance. In this work, the 4,4′-diamino-2,2′-biphenyldicarboxylic acid (DCB) or 3,5-diaminobenzoic acid (DABA) comonomer was incorporated into the 6FDA-TAPA network polyimide (PI) precursor via copolymerization. The molar content and the type of carboxylic-functionalized diamine were varied in order to easily tune the resulting network PI precursor structure. Then, these network PIs containing carboxyl groups underwent further decarboxylation crosslinking during the following heat treatment. Properties involving thermal stabilities, solubility, *d*-spacing, microporosity, and mechanical properties were investigated. Due to the decarboxylation crosslinking, the *d*-spacing and the BET surface areas of the thermally treated membranes were increased. Moreover, the content of DCB (or DABA) played a key role in determining the overall gas separation performance of the thermally treated membranes. For instance, after the heating treatment at 450 °C, 6FDA-DCB:TAPA (3:2) showed a large increment of about ~532% for CO_2_ gas permeability (~266.6 Barrer) coupled with a decent CO_2_/N_2_ selectivity~23.6. This study demonstrates that incorporating the carboxyl-containing functional unit into the PI backbone to induce decarboxylation offers a practical approach with which to tailor the micropore structure and corresponding gas transport properties of 6FDA-based network PIs prepared by the in situ crosslinking method.

## 1. Introduction

Membrane separation technology possesses the merits of low energy consumption, small footprints, and an eco-friendly nature for solving future energy-intensive separations such as natural gas purification, N_2_/O_2_ enrichment, He recovery, etc. [1,2,3,4]. Polyimide (PI), especially 4,4′-(Hexafluoroisopropylidene) diphthalicanhydride (6FDA)-based PI, is a promising candidate for gas separation owing to its high gas selectivity, remarkable chemical and thermal stability, and wide variety [5,6,7]. Commonly, permeability and selectivity represent two important parameters to describe its overall separation performance. Nevertheless, the PI membrane has usually been restricted to a trade-off effect that was proposed by Robeson and then theoretically explained by Freeman [8,9]. Moreover, the PI membrane has frequently been challenged due to the tricky plasticization issue, which can seriously affect the long-term stability of membrane operation on the industrial scale [10,11,12,13].

To address these issues, various strategies have been investigated thus far, and crosslinking is considered as an efficient method [14,15]. Briefly, the advantages of crosslinking can be summarized as follows: (1) Crosslinking can easily regulate the PI interchain distance and polymer rigidity that play key roles in determining the membrane’s gas separation performance according to Freeman’s theory [16,17,18]. (2) After crosslinking, the formed network PI usually has an improved anti-plasticization property, which is extremely significant for real separation occasions involving gas/VOC (volatile organic compound) mixtures. (3) In addition, the highly stable crosslinked structures enable these network PIs to have outstanding chemical and thermal stability [19,20]. On account of these reasons, network PIs have attracted significant attention for gas separation.

To date, two main methods have been applied to construct the network structures within the PI membrane: post-crosslinking and in situ crosslinking. In fact, the post-crosslinking method has been widely investigated by numerous researchers, and it generally refers to the creation of a covalent bond either induced via heating or formed using crosslinking agents such as diol and diamine [21,22,23,24,25]. For example, Kraftschik et al. used poly(ethylene glycol) to crosslink a 6FDA-DAM:DABA (3:2) PI membrane via an esterification reaction. The results proved that the resultant membrane’s anti-plasticization ability against acidic gas mixture was significantly enhanced [26]. In contrast, the in situ crosslinking method often uses multifunctional monomers (the number of functional groups is greater than two) in a polycondensation process to directly build the network structure within the PI without any additional post-processing steps. Recently, Jin’s group developed a triptycene-based network PI using 2, 6, 14-triaminotriptycene as the building block. They showed that the prepared network PIs possessed an outstanding molecular sieving property for N_2_/cyclohexane separation, with a rejection higher than 99% and N_2_ permeability of approximately 2000~2600 Barrer. However, the gas transport properties of other typical gas pairs such as CO_2_/N_2_, O_2_/N_2_, and CO_2_/CH_4_ for triptycene-based network PIs were not reported.

Recently, we successfully constructed a series of network PIs via the in situ crosslinking method using commercial 1,3,5-tris(4-aminophenyl) benzene (TAPB) or tris(4-aminophenyl)amine (TAPA) as the building block [27,28]. However, the gas permeabilities of these prepared network PI membranes were relatively low because of their tightly crosslinked structures. For example, the CO_2_ permeability of 6FDA-TAPA was only 37.4 Barrer, with the ideal CO_2_/N_2_ selectivity of ~32.8 [28]. As is well-known, the tailoring of the micropore structure (mainly involving an appropriate micropore size and narrow size distribution) within the PI is decisive for achieving a high gas separation performance. Accordingly, we paid great attention to the optimization of the amine monomer structure on the molecular scale, aiming to strengthen the corresponding network PI’s rigidity and thus create a more microporous PI membrane. Truxene triamine with a bulky pendant group, binaphthyl-ether tetramine, symmetrical “H-shaped” tetramine, and spirobifluorene pentamine were successively developed and used for reactions with 6FDA via the in situ crosslinking method in our previous studies [29,30,31,32]. As expected, the resulting network PI membranes all possessed superior overall gas transport properties relative to our previously reported 6FDA-TAPA and 6FDA-TAPB. For instance, the optimal network PI membrane derived from the “H-shaped” tetramine, termed as 6FDA-OHTA, possessed the CO_2_ permeability of ~225 Barrer with a corresponding CO_2_/N_2_ selectivity of ~30.4 [31]. Despite this, the synthetic routes of the above-mentioned new amine monomers were complicated and time-consuming. Moreover, these synthetic processes inevitably involved the use and discharge of toxic and hazardous solvents. Therefore, a facile strategy that could be used to tailor the micropore structure within the network PI membrane prepared by the in situ crosslinking method and, at the same time, regulate its corresponding gas transport property is still desired and worth studying.

Decarboxylation crosslinking, which was initially proposed by Koros’ group in the early 2000s, mainly refers to the thermal treatment of PI membranes containing carboxylic acid groups at the targeted temperatures [33,34,35,36,37]. In general, a rigid biphenyl linkage with stable C-C bonds was formed between PI segments during decarboxylation crosslinking, thus enhancing its anti-plasticization ability [38]. In addition, the release of CO_2_ during decarboxylation could expand the interchain distance and improve the gas permeabilities. For instance, after the decarboxylation-induced thermal crosslinking treatment, these crosslinked membranes displayed a notable anti-plasticization property at 1000 psia for CO_2_/CH_4_ mixed gas. Moreover, a remarkable increase in CO_2_ permeability was obtained for the crosslinked 6FDA-DAM:DABA (3:2) membrane.

Considering these advantages, decarboxylation was attempted to tailor the micropore structure of the network PI via copolymerization with a diamine monomer containing a thermally labile pendant carboxyl group. In detail, 4,4′-diamino-2,2′-biphenyldicarboxylic acid (DCB) containing two carboxyl groups and 3,5-diaminobenzoic acid (DABA) containing a single carboxyl group, respectively, were incorporated into the previous reported 6FDA-TAPA network PI backbone using copolymerization. Notably, the molar content and the type of the carboxylic-functionalized diamine were varied to tune the resulting network PI precursor structure conveniently. Then, these newly designed network PIs underwent further decarboxylation crosslinking during the following thermal treatment. Next, a systematic study focusing on the chemical structures, thermal stability, and the microporosity of the network PIs was carried out. In addition, pure gas and mixed gas permeation tests were conducted to study their overall gas transport properties. This study demonstrates that incorporating the carboxyl-containing functional unit into the PI precursor to induce decarboxylation provides a practical approach with which to tailor the micropore structure and corresponding gas separation performance of 6FDA-based network PIs prepared by in situ crosslinking reactions.

## 2. Experimental

### 2.1. Materials and Chemicals

In this study, 4,4′-(Hexafluoroisopropylidene) diphthalicanhydride (6FDA), Tris (4-aminophenyl) amine (TAPA), and 3,5-diaminobenzoic acid (DABA) were obtained from China Tech (Tianjin) Chemical Co., Ltd. The 4,4′-diamino-2,2′-biphenyldicarboxylic acid (DCB, 98%) was purchased from Aladdin Chemicals (Beijing). N, N-Dimethylformamide (DMF) was purchased from Tianjin Fu Chen Chemical Reagents Factory (Tianjin, China). DMF was purified by vacuum distillation and with molecular sieves before use. The gases with purities higher than 99.99% were purchased from Beijing Gas Company.

### 2.2. Synthesis of PI, Membrane Preparation, and Thermal Crosslinking

**Scheme 1:**
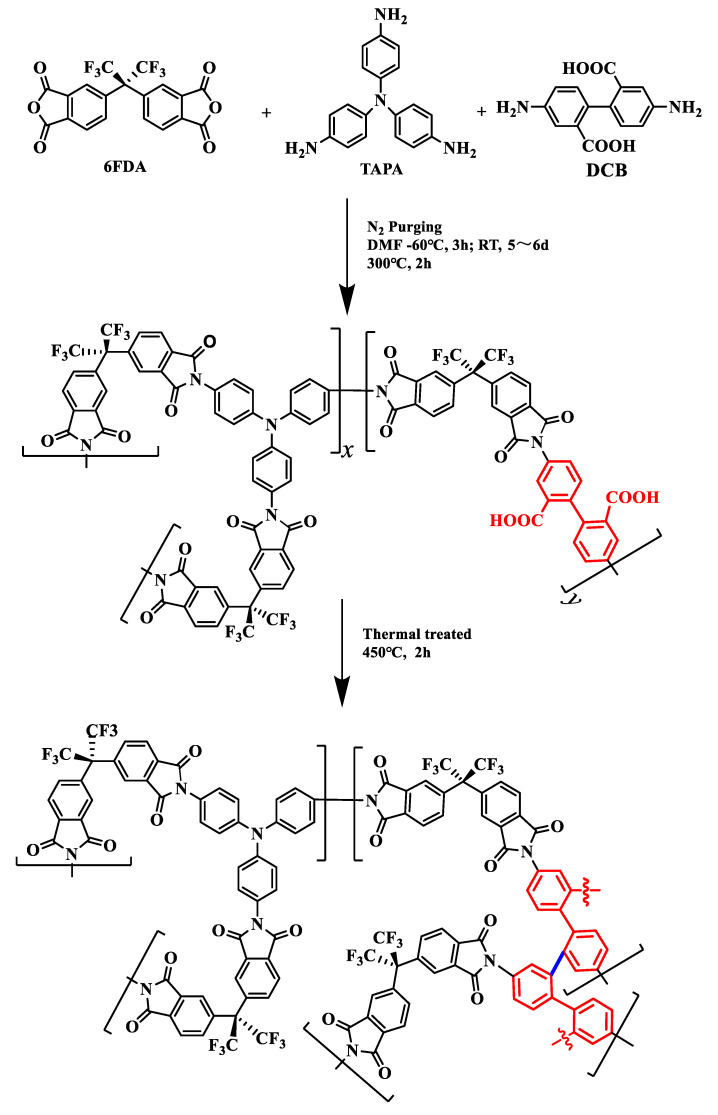
Synthesis of network PI precursors and their corresponding thermally treated membranes with different molar ratios of DCB and TAPA.

As shown in Scheme 1 and Scheme S1, a series of 6FDA-based network PIs were obtained by polycondensation reaction with predetermined molar ratios of DCB (or DABA) and TAPA. Notably, the molar ratio of amine functional groups to anhydride functional groups was always maintained at 1:1. The four prepared network PI precursors were named as 6FDA-DCB:TAPA (3:2), 6FDA-DCB:TAPA (1:1), 6FDA-DCB:TAPA (2:3), and 6FDA-DABA:TAPA (3:2), respectively, according to their monomer compositions. The synthesis procedure for 6FDA-DCB:TAPA (3:2) is described as an example. Specifically, DCB (0.163 g, 0.60 mmol) and TAPA (0.116 g, 0.40 mmol) were added into a Schlenk flask. In an argon atmosphere, DMF (22 mL) was added, and then the resulting solution was stirred at -60 °C for 15 min. Into this solution, 6FDA (0.533 g, 1.20 mmol) dissolved in 5 mL of DMF was slowly dripped through a syringe and stirred for another 3 h to obtain the corresponding poly(amic acid) (PAA) solution, and then this solution was poured into a leveled Petri dish. After slow solvent evaporation for 5 days at 50 °C, a dense membrane was obtained. Then, the membrane was further heated at 80 °C for 2 days to remove any residual solvent. Finally, the prepared membrane was thermally imidized at 300 °C for 2 h (at a heating rate of 2 °C·min^−1^). Using a similar method, several other network PI precursors (6FDA-DCB:TAPA (1:1), 6FDA-DCB:TAPA (2:3), and 6FDA-DABA:TAPA (3:2)) were prepared.

Next, the following heating treatment was conducted using a tube furnace (SK-G06123K, Tianjin ZhongHuan Experiment Electric Furnace CO., Ltd., Tianjin, China): Specifically, the prepared network PI precursors were placed between two porous graphite sheets to induce decarboxylation crosslinking at the target temperature for 2 h in a N_2_ atmosphere. The annealing conditions were determined according to the Thermo gravimetric analysis (TGA) results of the network PI precursors. Heating temperatures of 400, 430, and 450 °C were selected. The thermally treated network PI membranes were named based on their heating temperatures. For instance, 6FDA-DCB:TAPA (3:2)-450 means that 6FDA-DCB:TAPA (3:2) was thermally treated at 450 °C for 2 h.

### 2.3. Characterizations

The Fourier transform infrared (FT-IR) spectra of the PI precursors and their corresponding thermally treated membranes were obtained using a Perkine Elmer 782 Fourier transform spectrophotometer. The surfaces and cross-sectional morphologies of the samples were identified via a high-resolution field-emission scanning electron microscope (SEM, Zeiss, Jena, Germany). Wide-angle *X*-ray diffractometry (WAXD) was recorded on a Rigaku Ultima IVX-ray Diffractometer. The average *d*-spacing was obtained according to Bragg’s law. TGA was performed using a TA instruments Q500. The heating rate of TGA was 10 °C min^−1^. The mechanical properties were obtained using an electronic universal material testing machine (Shenzhen SUNS Technology Stock Co., Ltd. (Shenzhen, China), UTM6103, maximum test force 1 kN). The CO_2_ adsorption behaviors of the samples were measured through adsorption experiments at 77 K using a Micromeritics ASAP 2460 2.02 Surface Area and Porosity Analyzer.

### 2.4. Gas Separation Performance Measurements

All the gas separation tests were conducted in the order of N_2_, O_2_, and CO_2_ at 30 °C at a feeding pressure of 0.2 MPa according to the constant-volume/variable-pressure approach. The permeation setup was custom-made, and its flow chart for a single gas permeation test is shown in Figure S1a. The feeding pressure varied from 0.2 MPa to 1.2 MPa, aiming to elucidate the effect of feeding pressure on PI membranes’ gas transport properties. The permeability (*P*, Barrer) was calculated from the growth rate of the steady-state pressure increment downstream (*dp*/*dt*, cm Hg s^−1^) according to Equation (1):

(1) $$P=\frac{273}{76}\cdot \frac{V_{d}\cdot l}{A\cdot T\cdot p_{up}}\cdot (\frac{dp}{dt})$$

where *P* (Barrer, 1 Barrer = 10^−10^ cm^3^ (STP) cm cm^−2^ s^−1^ cmHg^−1^) refers to the gas permeability, *V_d_* is the calibrated downstream volume (cm^3^), *l* corresponds to the membrane thickness (cm), *p_up_* is the upstream pressure (cm Hg), *A* is the effective area (cm^2^), and *T* is the temperature (K).

The ideal selectivity was defined as the ratio of permeability for gas A and gas B using the following Equation (2):

(2) $$\alpha =\frac{P_{A}}{P_{B}}$$

The diffusion coefficient (*D*) of the network PI membrane was calculated using Equation (3):

(3) $$D=\frac{l^{2}}{6\theta }$$

where *θ* is the diffusion time lag. The solubility coefficient (*S*) was estimated according to the following Equation (4):

(4) $$S=\frac{P}{D}$$

The diffusivity selectivity ($\alpha_{D}$) and solubility selectivity ($\alpha_{S}$) were obtained based on the following relationships:

(5) $$\alpha_{D}=\alpha_{A/B}=\frac{D_{A}}{D_{B}}$$

(6) $$\alpha_{S}=\alpha_{A/B}=\frac{S_{A}}{S_{B}}$$

The mixed gas separation of the network PI membrane was obtained using the binary gas mixture with 15% CO_2_ and 85% N_2_, and the testing diagram is shown in Figure S1b. H_2_ was used as the sweep gas, and the gas concentration of each component was determined by gas chromatography (GC). The gas permeability of each component can be expressed based on Equation (7). Similarly, the mixed gas selectivity of CO_2_/N_2_ was also calculated according to Equation (2):

(7) $$P_{i}=\frac{y_{i}\cdot V_{d}\cdot l}{x_{i}{\cdot p}_{up}\cdot T\cdot R\cdot A}\cdot \frac{dp}{dt}$$

where *P_i_* (Barrer, 1 Barrer = 10^−10^ cm^3^ (STP) cm cm^−2^ s^−1^ cmHg^−1^) is the gas permeability of component *i*, *x_i_* and *y_i_* are the individual gas (*i*) component of the feed and permeate side, respectively, and *R* = 0.278 (cm^3^ cmHg cm^−3^(STP) K^−1^).

## 3. Results and Discussions

### 3.1. Synthesis of Network PIs and Structural Transformation during Heating Treatment

As shown in Scheme 1, the molar ratio of the dual-carboxylic-functionalized DCB and TAPA was changed to regulate the degree of the following decarboxylation crosslinking within the copolymer. The molar ratio of DABA: TAPA was fixed at 3:2 in the present work, which was the preferred molar ratio based on the overall gas transport property results of the 6FDA-DCB: TAPA membrane. The synthetic route of the four network PI precursors involved two steps, namely, the PAA preparation and subsequent imidization process carried out by heating treatment. This method is highly consistent with the traditional two-step method for linear PI preparation. It is worth noting that the tridimensional polycondensation, which involved dianhydride (6FDA) and triamine (TAPA), was difficult to control due to the high risk of gelation from the perspective of Flory’s theory. In order to solve this problem, a low-temperature preparation strategy was used to regulate the initial reactivity between the anhydride and amino functional groups during polycondensation. A specific description of the low-temperature synthetic strategy is provided in our prior studies [27,28].

**Figure 1:**
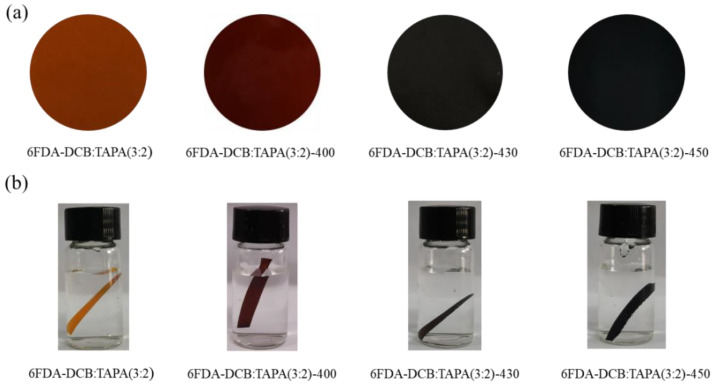
Optical pictures (**a**) and solubility tests (**b**) of 6FDA-DCB: TAPA (3:2) and its thermally treated membranes at 400 °C, 430 °C, and 450 °C for 2 h.

The optical pictures of the prepared network PIs and their thermally treated membranes are shown in Figure 1a and Figure S2. It could be observed that the color of the network PI membranes became increasingly darker as the temperature increased from 400 to 450 °C. FE-SEM was used to examine the membrane surfaces and their cross-sections. As displayed in Figure S3, each obtained network PI film, with a thickness of about 50 μm, has a smooth and dense morphology without clear flaws. Then, all the films were soaked in 80 °C DMF solution for 96 h in order to conduct the solubility tests, and the results are shown in Figure 1b and Figure S4. As expected, all the prepared network PI precursors and their thermally treated membranes were insoluble in DMF before and after heat treatment because of their crosslinked structures within the polymer. In addition, the gel contents of 6FDA-DCB:TAPA (3:2) were calculated. As depicted in Figure S5, negligible weight loss was observed for 6FDA-DCB:TAPA (3:2), exhibiting its superior organic solvent resistance.

**Figure 2:**
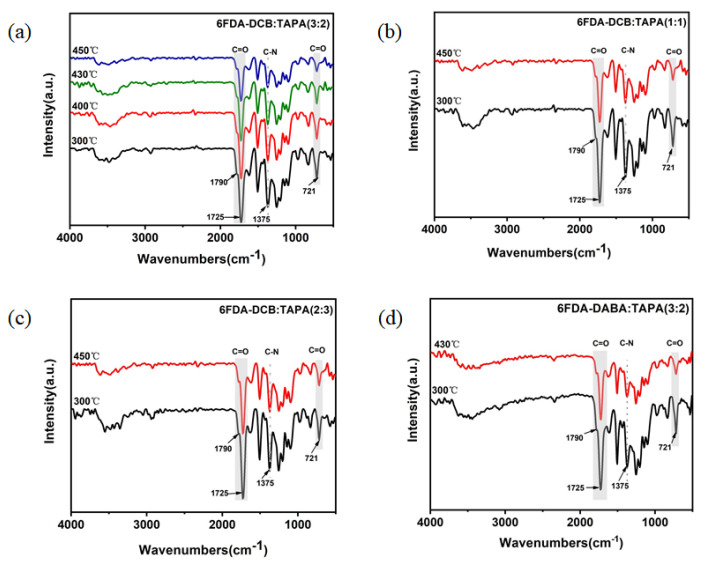
FT-IR spectra of network PI precursors: (**a**) 6FDA-DCB:TAPA (3:2) (**b**) 6FDA-DCB:TAPA (1:1) (**c**) 6FDA-DCB:TAPA (2:3) (**d**) 6FDA-DABA:TAPA (3:2) and their corresponding membranes which were thermally treated at different temperatures.

The variation in the chemical structures for the four network PI precursors and their corresponding membranes, which were thermally treated at different temperatures, were examined using FT-IR spectra, and the results are summarized in Figure 2. Concerning the four network PI precursors including 6FDA-DCB:TAPA (3:2), 6FDA-DCB:TAPA (1:1), 6FDA-DCB:TAPA (2:3) and 6FDA-DABA:TAPA (2:3), the typical absorption bands of imide groups can be identified at 1790 cm^−1^ (C=O, asymmetric stretching), 1725 cm^−1^ (C=O, symmetric stretching), 1375 cm^−1^ (C-N, stretching vibration), 721 cm^−1^ (C=O, bending vibration). Additionally, the feature peak of the PAA proton in the scope of 1620–1680 cm^−1^ almost disappeared, showing that the PAA were completely imidized.

In addition, a broad peak at 3700–3200 cm^−1^ could be identified for the four network PI precursors. This phenomenon is mainly ascribed to the -OH from the carboxyl groups and demonstrates the existence of hydrogen bonds in the four network PI precursors. However, after the heat treatment at the target temperatures, the intensity of this broad peak was clearly weakened in the case of 6FDA-DCB:TAPA (3:2)-450, 6FDA-DCB:TAPA (1:1)-450, 6FDA-DCB:TAPA(2:3)-450 and 6FDA-DABA:TAPA (2:3)-430. According to the previously reported decarboxylation mechanism of PI containing carboxyl groups, two neighboring carboxyl groups from DCB or DABA units yield an anhydride during heat treatment, and then this anhydride decomposes through the release of CO_2_ to produce two phenyl radicals under sufficient high-temperature conditions. Finally, two adjacent phenyl radicals are combined to form the crosslinking biphenyl linkage [33,34,35]. In view of this, the disappearance of the broad peak at 3200–3700 cm^−1^ was mainly due to the removal of the carboxyl groups, along with the process of decarboxylation crosslinking.

The structural transformation of 6FDA-DCB:TAPA (3:2) was further investigated at the different treatment temperatures of 400 °C, 430 °C, and 450 °C. As shown in Figure 2a, the hydrogen bonds within the thermally treated network PI gradually became weaker with the increase in the treatment temperature from 400 °C to 450 °C. It further proved that the degree of decarboxylation crosslinking within the thermally treated PI membrane was closely related to the heating temperature. Additionally, the typical peaks of the imide group at 1790, 1725, 1375, and 721 cm^−1^ showed that the main polymer chain of the network PI still existed after the thermal treatment.

### 3.2. Thermal Properties

**Figure 3:**
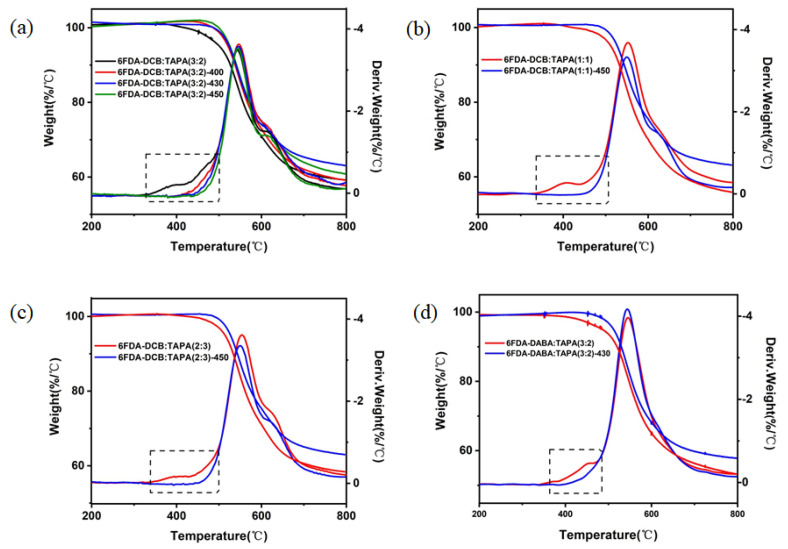
TGA curves of network PI precursors: (**a**) 6FDA-DCB:TAPA (3:2) (**b**) 6FDA-DCB:TAPA (1:1) (**c**) 6FDA-DCB:TAPA (2:3) (**d**) 6FDA-DABA:TAPA (3:2) and their corresponding membranes which were thermally treated at different temperatures.

In general, the slight weight loss in the TGA curve and the corresponding smaller peak in the derived weight curve at the lower temperature prior to the degradation of the main PI backbone is the typical feature of decarboxylation crosslinking of PI containing carboxylic function groups. Figure 3 shows the TGA curves as well as the derivative weight curves for all four network PI precursors and their corresponding membranes that were thermally treated at different targeted temperatures. As shown in the figure, the four network PI precursors yielded similar curves, i.e., 6FDA-DCB:TAPA (3:2), 6FDA-DCB:TAPA (1:1), 6FDA-DCB:TAPA (2:3) and 6FDA-DABA:TAPA (3:2) exhibited two-stage weight loss, with the first stage ranging from 330 to 500 °C and the second stage starting at approximately 500 °C. A similar phenomenon has been widely reported by other researchers for linear PI containing a carboxylic pendant group [34,35,36,37]. Obviously, the first minor weight loss represents the removal of carboxylic groups in DCB or DABA moieties, and it also further indicates that the decarboxylation of the carboxylic groups starts at approximately 330 °C. On the other hand, the second weight loss stage was ascribed to the main PI backbone’s degradation. 

In contrast, regarding the thermally treated membranes (6FDA-DCB:TAPA (3:2)-450, 6FDA-DCB:TAPA (1:1)-450, 6FDA-DCB:TAPA (2:3)-450 and 6FDA-DABA:TAPA (3:2)-430), significant weight loss was identified only for the main network PI backbone subjected to degradation at high temperatures, while the weight loss rooting from decarboxylation process was almost vanished. Furthermore, a detailed TGA curve and derivative weight curve for 6FDA-DCB:TAPA (3:2) at different heating temperatures were obtained. As shown in Figure 3a, the intensity of the first minor degradation gradually became weaker as the heating temperature rose and eventually vanished for the membranes thermally treated at 450 °C for 2 h. In fact, these results are in good agreement with the FTIR spectroscopy results, as shown in Figure 2a, indicating that the intensity of the broad peaks at 3200–3700 cm^−1^ originating from the carboxylic group in the DCB moiety became weaker for the thermally treated 6FDA-DCB:TAPA (3:2) as the heating temperature increased from 400 °C to 450 °C. In view of the above discussion, we can infer that the removal of carboxyl groups was almost complete when the network PI precursors were treated under our target thermal conditions.

As shown in Table 1, the four designed network PI precursors had extremely high decomposition temperatures (e.g., *T*_d,5%_ = 499–527 °C), exhibiting their excellent thermal stability. The greater DCB component in the PI backbone indicated the intensified hydrogen bond effect resulting from more carboxylic groups; thus, the greatest rigidity was obtained for the 6FDA-DCB:TAPA (3:2) PI precursor with the highest DCB content.

### 3.3. Polymer Chain Packing, Microporosity, and Mechanical Properties 

**Figure 4:**
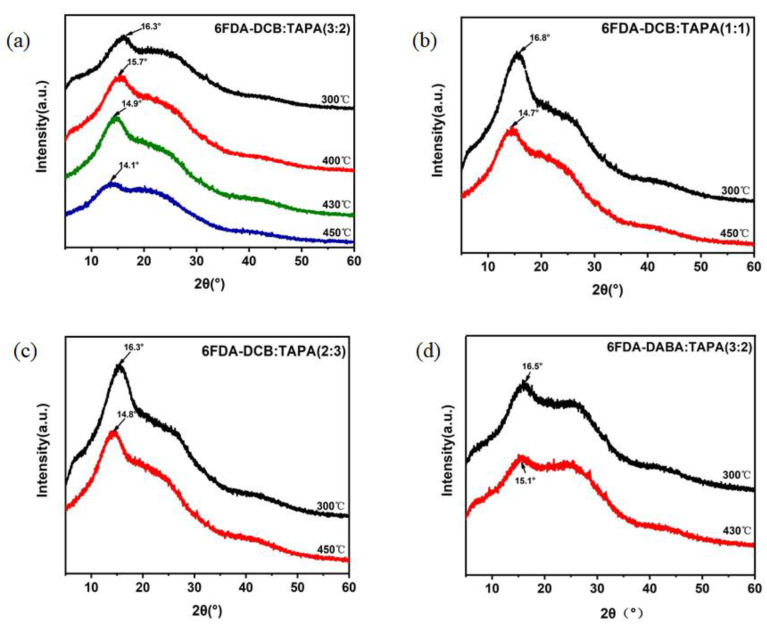
Wide-angle X-ray diffraction (WAXD) analysis of four network PI precursors: (**a**) 6FDA-DCB:TAPA (3:2) (**b**) 6FDA-DCB:TAPA (1:1) (**c**) 6FDA-DCB:TAPA (2:3) (**d**) 6FDA-DABA:TAPA (3:2) and their corresponding membranes which were thermally treated at different temperatures.

WAXD was employed to investigate the variation trend of average interchain spacings for the network PI precursors and their corresponding thermally treated membranes. Figure 4 shows their WAXD patterns, and the relevant *d*-spacing values obtained using Bragg’s law are listed in Table 1. As expected, all the prepared network PI membranes displayed broad peaks owing to their amorphous structures. Compared with the PI precursor, the 2*θ* value of each thermally crosslinked membrane shifted to the left, i.e., a smaller diffraction angle was observed. This meant that the thermally treated membranes possessed larger *d*-spacing values than their precursors because of the decarboxylation crosslinking. Obviously, the decarboxylation crosslinking within the PI membrane could release the CO_2_ and create additional free volume in the thermally treated membranes, and thus a higher *d*-spacing value was attained. For instance, the thermal treatment of 6FDA-DCB:TAPA (3:2) resulted in gradually increased *d*-spacing values of 5.64 Å (400 °C), 5.94 Å (430 °C), and 6.28 Å (450 °C) due to the increasing degree of decarboxylation crosslinking. Moreover, as shown in Figure 4a–c, with the increasing DCB molar content, the *d*-spacing value rose from ~5.98 Å for 6FDA-DCB:TAPA (2:3)-450 to ~6.28 Å for 6FDA-DCB:TAPA (3:2)-450 under the same heat treatment conditions. These results indicated that a more cross-linkable DCB moiety within the network PI precursor could create larger interchain distances due to the higher degree of decarboxylation crosslinking after the thermal treatment. 

**Figure 5:**
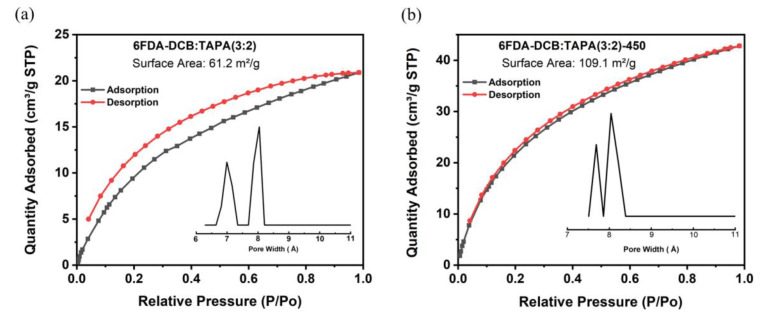
Physisorption isotherms for 6FDA-DCB:TAPA (3:2) (**a**) and 6FDA-DCB:TAPA (3:2)-450 (**b**) using CO_2_ at 0 °C.

Figure 5 shows the CO_2_ adsorption/desorption isotherms, pore size distributions (PSD), and BET surface areas of 6FDA-DCB:TAPA (3:2) and 6FDA-DCB:TAPA (3:2)-450. Compared to the 6FDA-DCB:TAPA (3:2) precursor, the BET surface areas increased from 61.2 m^2^/g to 109.1 m^2^/g after heat treatment at 450 °C for 2 h. Apparently, the increased BET surface areas represent a larger free volume within the network PI membrane after heat treatment. 

Furthermore, Figure S6 and Table S1 display the mechanical properties of our prepared samples, 6FDA-DCB:TAPA (3:2) and 6FDA-DCB:TAPA (3:2)-450. As shown, the 6FDA-DCB:TAPA (3:2) precursor possessed a high tensile strength of ~131 MPa coupled with reasonable elongation at break value of ~5.6%. However, both the tensile strength and elongation at break clearly decreased after the heat treatment at 450 °C for 2 h. In detail, the tensile strength decreased to 38 MPa, while the elongation at break decreased from ~5.6% to ~2.2% for 6FDA-DCB:TAPA (3:2)-450. This meant that the thermally treated PI membranes became more brittle. Although this changing trend of the mechanical properties has been widely reported in other 6FDA-based PI membranes such as PI-Im-COOH-450-2 and 6FDA-DAM_0.8_-DCB_0.2_-400, we found that, at the same temperature, the overall mechanical properties of our prepared 6FDA-DCB:TAPA (3:2)-450 membrane were slightly worse. Since most reported PI precursors have a linear structure, the present 6FDA-DCB:TAPA (3:2) precursor was already a crosslinked structure with high rigidity. Thus, after the heat treatment at 450 °C for 2 h, the overall mechanical properties of 6FDA-DCB:TAPA (3:2)-450 were worse than those of thermal decarboxylation membranes, resulting from the linear PI precursor.

### 3.4. Pure Gas Separation Properties

Table 2 lists the gas permeabilities and ideal selectivity data of the prepared network PI membranes before and after thermally induced decarboxylation crosslinking for N_2_, O_2_, and CO_2_ at 30 °C under a pressure of 2 bar. As shown, the sequence of gas permeability for all the tested membranes is *P*(N_2_) < *P*(O_2_) < *P*(CO_2_) as a function of the reversed kinetic diameter order of the gas molecules. This result proved that diffusivity dominates the gas separation performance of the network PI membrane. Prior studies have shown that the crosslinking temperature plays a key role in determining the membrane’s gas separation performance during decarboxylation crosslinking [36]. The effect of the heating temperature on the gas transport properties of the 6FDA-DCB:TAPA (3:2) PI precursor were investigated, and the results are listed in Table 2. Because of the gradually increasing degree of decarboxylation crosslinking, the gas permeability was clearly enhanced as the treatment temperature rose. This is mainly attributed to the fact that decarboxylation crosslinking can create a more open matrix and larger free volume within the PI; thus, enhanced CO_2_ permeabilities were achieved. As a result, 6FDA-DCB:TAPA (3:2)-450 possessed the largest CO_2_ gas permeability of ~266.6 Barrer coupled with a decent CO_2_/N_2_ selectivity of ~23.6.

According to the above promising results, the other 6FDA-DCB:TAPA (1:1) and 6FDA-DCB:TAPA (2:3) PI precursors were only investigated at the optimal temperature of 450 °C for 2 h. On the other hand, considering the degradation temperature of the PI backbone (starting at about 500 °C) and the decarboxylation, which proceeded based on the above-mentioned TGA results, a further increase in the heating temperature was not investigated in the present work. Notably, the thermal treatment temperature adopted for 6FDA-DABA:TAPA (3:2) was 430 °C instead of 450 °C, as the thermally crosslinked 6FDA-DABA:TAPA (3:2)-450 membrane was quite fragile and thus could not withstand the gas separation testing. As a result, all the crosslinked membranes treated at 450 °C exhibited evidently improved gas permeabilities with acceptable decreases in their corresponding selectivities. In detail, the thermal treatment of the network PI precursor at 450 °C enabled sharp increases in the CO_2_ permeabilities by 532%, 417%, 382%, and 495% for 6FDA-DCB:TAPA (3:2), 6FDA-DCB:TAPA (1:1), 6FDA-DCB:TAPA (2:3) and 6FDA-DABA:TAPA (3:2), respectively. At the same time, the pure gas CO_2_/N_2_ selectivity decreased by 21.1%, 20.7%, 14.6%, and 2.1%, respectively, for the corresponding thermally crosslinked PI membranes. Moreover, concerning the network PI precursor containing the DCB moiety, we also found that a more cross-linkable DCB moiety in the PI precursor commonly led to a larger increment in gas permeability after thermal treatment due to the higher degree of decarboxylation crosslinking within the PI precursor, a finding which is also supported by the above-mentioned comparison results of the *d*-spacing values.

To understand the effect of decarboxylation crosslinking on gas separation performance in detail, the solubility coefficient (*S*), the diffusion coefficient (*D*), the solubility selectivity (*α*_D_), and the diffusion selectivity (α_S_) are displayed in Table S2. The results demonstrate that the increase in gas permeability after the thermal treatment at 450 °C for 2 h was mainly due to the fast growth of the *D* value rather than the *S* value, especially for the O_2_ and N_2_. For instance, regarding 6FDA-DCB:TAPA (3:2)-450, the *D* value for O_2_ was improved by 356%, while the *S* value for O_2_ was only improved by 43%, in comparison to the values of 6FDA-DCB:TAPA (3:2). According to the solution–diffusion model (Equation (5)), *D* depends mainly on the gas kinetic diameter and free volume of the polymer matrix, while *S* depends on the condensability of permeable gases and the interactions between the gas and polymer. Since the decarboxylation crosslinking within the copolyimide could create additional free volume, the *D* value was clearly increased. In addition, the *S* values of CO_2_ were clearly larger than those of N_2_ and O_2_, since CO_2_ was the condensable gas.

### 3.5. Comparison with Upper Bound and Other Studies

**Figure 6:**
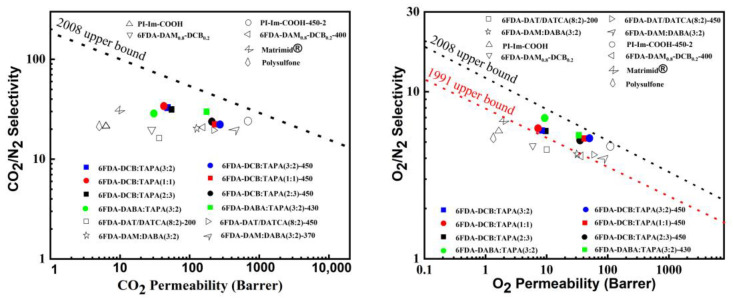
Relationship between permeability and selectivity of network PI precursors and their corresponding thermally treated membranes with respect to Robeson upper bounds.

Figure 6 shows the overall gas separation performance of the prepared network PI precursors and their thermally treated membranes together with other related state-of-the-art membranes for CO_2_/N_2_ and O_2_/N_2_ gas pairs and the upper bound lines proposed by Roberson for 1991 and 2008. As shown here, by incorporating the carboxyl-containing diamine unit to induce decarboxylation crosslinking, the overall gas transport properties of the thermally treated network PI membranes were enhanced, moving towards to the 2008 upper bound, especially for the O_2_/N_2_ gas pair. Additionally, we found that our prepared thermally treated membranes, including 6FDA-DCB:TAPA (3:2)-450, 6FDA-DCB:TAPA (1:1)-450, 6FDA-DCB:TAPA (2:3)-450, and 6FDA-DABA:TAPA (3:2)-430, had comparable or even superior overall gas transport properties to Matrimid^®^ [39], Polysulfone [39], 6FDA-DAM_0.8_-DCB_0.2_-400 [33] and 6FDA-DAT/DATCA(8:2)-450 [40]. Meanwhile, compared with 6FDA-DAM:DABA (3:2)-370 [41] and PI-Im-COOH-450-2 [34], the gas permeabilities of these thermally treated network PI membranes were lower. This phenomenon was mainly attributed to the tightened polymer chains originating from the crosslinked 6FDA-TAPA moiety compared to the linear PI moieties, such as 6FDA-DAM and PI-Im.

**Table 2:** Pure gas permeabilities and selectivities for the network PI precursors and their membranes thermally treated at different temperatures.

PIs	*P* (Barrer)			α		
	N_2_	O_2_	CO_2_	O_2_/N_2_	CO_2_/N_2_	
6FDA-DCB:TAPA (3:2)	1.41 ± 0.1	8.78 ± 0.8	42.18 ± 3.1	6.2 ± 0.5	29.9 ± 1.9	
6FDA-DCB:TAPA (3:2)-400	2.61 ± 0.3	15.79 ± 1	57.07 ± 2.8	6.1 ± 0.4	21.9 ± 1.4	
6FDA-DCB:TAPA (3:2)-430	4.26 ± 0.4	21.19 ± 1.3	88.25 ± 3.5	5 ± 0.2	20.7 ± 1.1	
6FDA-DCB:TAPA (3:2)-450	11.32 ± 0.7	57.29 ± 3	266.6 ± 6.5	5.1 ± 0.1	23.6 ± 0.9	
6FDA-DCB:TAPA (1:1)	1.58 ± 0.2	9.23 ± 0.6	46.48 ± 2.8	5.8 ± 0.3	29.4 ± 1.8	
6FDA-DCB:TAPA (1:1)-450	10.33 ± 0.8	49.71 ± 2.4	240.18 ± 5.9	4.8 ± 0.2	23.3 ± 1.2	
6FDA-DCB:TAPA (2:3)	1.72 ± 0.2	10.21 ± 0.9	48.76 ± 2.7	5.9 ± 0.2	28.3 ± 1.4	
6FDA-DCB:TAPA (2:3)-450	9.52 ± 0.7	46.42 ± 2.6	235.13 ± 6.1	4.9 ± 0.1	24.7 ± 1.1	
6FDA-DABA:TAPA (3:2)	1.39 ± 0.1	10.17 ± 0.5	34.2 ± 3.6	7.3 ± 0.2	28.2 ± 2	
6FDA-DABA:TAPA (3:2)-430	7.37 ± 0.6	39.6 ± 2.5	203.45 ± 6.2	5.4 ± 0.2	27.6 ± 1.3	
6FDA-DAT/DATCA (8:2)-200	2.4	10.5	40.4	4.4	16.8	[40]
6FDA-DAT/DATCA (8:2)-450	12.6	58.1	241.2	4.6	19.1	[40]
6FDA-DAM:DABA (3:2)	7.04	32	144	4.5	20.5	[41]
6FDA-DAM:DABA (3:2)-370	25.2	98	485.4	3.9	19.3	[41]
PI-Im-COOH	0.3	1.8	6.7	6	22.3	[34]
PI-Im-COOH-450-2	27.1	132.8	685.1	4.9	25.3	[34]
6FDA-DAM_0.8_-DCB_0.2_	1.45	7.08	32	4.9	22.1	[33]
6FDA-DAM_0.8_-DCB_0.2_-400	7.9	35	174	4.4	22	[33]
Matrimid^®^	0.32	2.1	10	6.6	31	[39]
Polysulfone	0.25	1.4	5.6	5.6	22.4	[39]

### 3.6. Effects of Feeding Pressure, Physical Aging Performance, and Mixed Gas Separation

To gain more insight into the gas transport properties of the prepared network PI membranes, the effects of the feeding pressure, physical aging, and mixed gas separation were studied for 6FDA-DCB:TAPA (3:2)-450 and 6FDA-DABA:TAPA (3:2)-430. According to the Robeson upper bound comparison results, 6FDA-DCB:TAPA (3:2)-450 possessed the best overall gas separation performance among the DCB-containing network PI membranes. Moreover, 6FDA-DABA:TAPA (3:2)-430 was also selected due to the fact that it had the best CO_2_/N_2_ selectivity among all the thermally crosslinked membranes.

**Figure 7:**
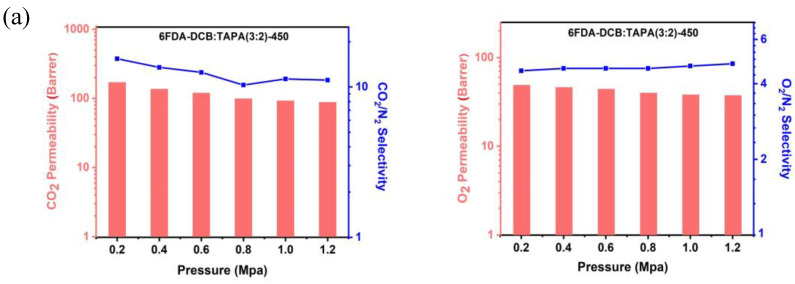

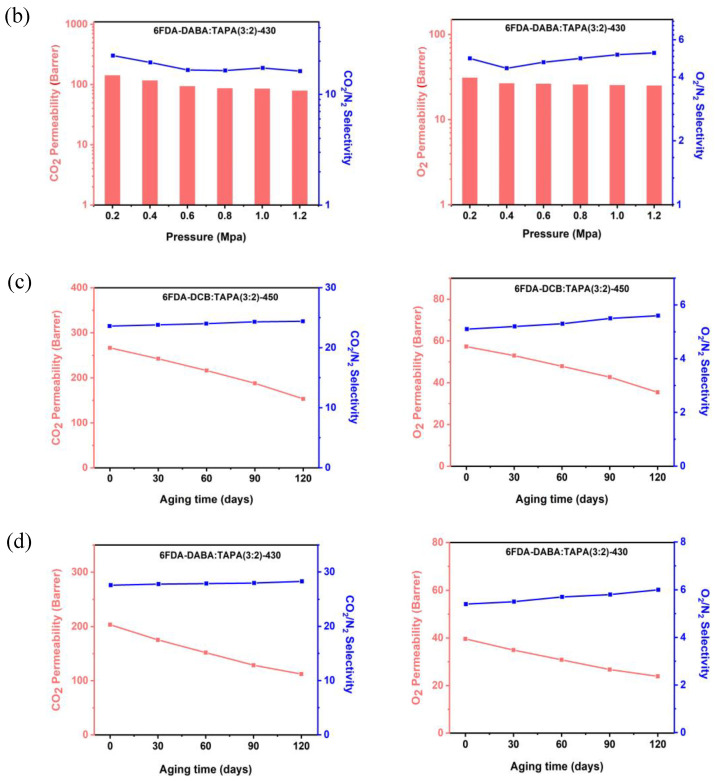
Pure gas permeabilities and ideal selectivities of 6FDA-DCB:TAPA (3:2)-450 and 6FDA- DABA:TAPA (3:2)-430 with different test pressures (**a**,**b**) at 30 °C and different aging times (**c**,**d**) at 0.2 MPa and 30 °C.

Figure 7a,b and Table S3 represent the pressure dependence of gas permeability and selectivity variation for 6FDA-DCB:TAPA (3:2)-450 and 6FDA-DABA:TAPA (3:2)-430. It was found that the gas permeabilities of both membranes decreased slightly with the upstream pressure increase. This phenomenon can be explained by the classic dual-mode sorption theory for mass transfer in glassy PI [42,43]. It is noteworthy that no obvious plasticization was observed in 6FDA-DCB:TAPA (3:2)-450 or 6FDA-DABA:TAPA (3:2)-430 as the CO_2_ pressure reached 1.2 MPa. Figure 7c,d and Table S4 display the changing trend of the separation performance over time for 6FDA-DCB:TAPA (3:2)-450 and 6FDA-DABA:TAPA(3:2)-430. Generally, physical aging leads to a decrease in gas permeability coupled with a slight increase in gas selectivity [31,32,34]. This rule was followed by our prepared 6FDA-DCB:TAPA (3:2)-450 and 6FDA-DABA:TAPA (3:2)-430. For instance, after being monitored for 120 days, the CO_2_ and N_2_ permeabilities of 6FDA-DCB:TAPA (3:2)-450 decreased from 266.6 Barrer and 11.32 Barrer to 153.35 Barrer and 6.29 Barrer, respectively. Meanwhile, the CO_2_/N_2_ selectivity increased slightly from 23.6 to 24.4.

Lastly, to study the mixed gas transport properties of the 6FDA-DCB:TAPA (3:2)-450 and 6FDA-DABA:TAPA (3:2)-430 membranes, the mixed CO_2_/N_2_ (15/85, *v*/*v*) gas pair was applied for an in-depth study. As shown in Table S5, 6FDA-DCB:TAPA(3:2)-450 had the CO_2_ permeability of ~230.7 Barrer coupled with CO_2_/N_2_ selectivity~23.9 that is very close to the pure gas separation performance. A similar phenomenon was found for 6FDA-DABA:TAPA (3:2)-430. These separation performance deviations between pure gas and mixed gas for the tested membranes can be explained by competitive sorption between CO_2_ and N_2_.

Based on the above discussion, we can infer that incorporating the carboxyl-containing functional unit into the PI precursor to induce decarboxylation was an effective approach for tailoring the micropore structure and corresponding gas separation performance of 6FDA-based network PIs prepared by the in situ crosslinking method. However, the extremely low synthetic temperature, ranging from -50 °C to -60 °C, leads to high energy consumption. In addition, the overall mechanical properties of prepared thermally treated PI membranes should be further improved.

## 4. Conclusions

In this work, a series of network copolyimide membranes containing a carboxyl functional group were obtained via the copolymerization method, and then these network PIs underwent further decarboxylation crosslinking at different heating temperatures to easily tailor the micropore structures and corresponding gas transport properties of the membranes. All the prepared network PI membranes possessed high polymer rigidity, excellent thermal stability, and limited solubility in DMF due to their firm crosslinked structures. After heat treatment at the target temperatures, the average interchain distance and microporosity of the thermally treated membranes were increased, as supported by the increased *d*-spacing and BET surface area values. As a result, thermal treatment of the network PI precursors enabled sharp increases in the CO_2_ permeabilities of 532%, 417%, 382%, and 495% for 6FDA-DCB:TAPA (3:2), 6FDA-DCB:TAPA (1:1), 6FDA-DCB:TAPA (2:3) and 6FDA-DABA:TAPA (3:2), respectively. At the same time, the pure gas CO_2_/N_2_ selectivity decreased by 21.1%, 20.7%, 14.6%, and 2.1%, respectively, for the corresponding thermally crosslinked PI membranes. The solubility and diffusion coefficients of all the prepared membranes indicate that the remarkably enhanced permeabilities were mainly attributed to the rapid growth in the *D* value. We believe that incorporating a carboxyl-containing functional unit in the PI backbone provides an effective method for adjusting the micropore structure and gas transport properties of network PIs prepared by the in situ crosslinking method.

## Figures and Tables

**Table 1:** Thermal properties, *T*_g_, 2*θ,* and *d-*spacing of network PI precursors and their membranes thermally treated at different heating temperatures.

PIs	*T*_d,5%_ (°C)	*T*_d,10%_ (°C)	2*θ* (°)	*d-*Spacing (Å)
6FDA-DCB:TAPA (3:2)	513.6	538.9	16.3	5.43
6FDA-DCB:TAPA (3:2)-400	521.4	538	15.7	5.64
6FDA-DCB:TAPA (3:2)-430	527.4	542.4	14.9	5.94
6FDA-DCB:TAPA (3:2)-450	499	553.1	14.1	6.28
6FDA-DCB:TAPA (1:1)	517.1	535.4	16.8	5.27
6FDA-DCB:TAPA (1:1)-450	509.4	533.7	14.7	6.02
6FDA-DCB:TAPA (2:3)	514.9	534.2	16.3	5.43
6FDA-DCB:TAPA (2:3)-450	510.3	537.5	14.8	5.98
6FDA-DABA:TAPA (3:2)	510.7	529.8	16.5	5.37
6FDA-DABA:TAPA (3:2)-430	519.4	535.1	15.1	5.86

## Data Availability

Data are contained within the article.

## Supplementary Materials

The following supporting information can be downloaded at: https://www.mdpi.com/article/10.3390/membranes13050461/s1, References [44,45,46,47] are cited in the Supplementary Material. Scheme S1. Synthesis of 6FDA-DABA:TAPA(3:2) network PI and its thermally treated membrane 6FDA-DABA:TAPA(3:2)-430. Figure S1. Flow chart of gas transport testing for (a) pure gas, (b) mixed gas. Figure S2. Optical pictures of prepared network PIs and their thermally treated membranes. Figure S3. SEM images of prepared network PIs and their thermally treated membranes. Figure S4. Solubility test of prepared network PIs and their thermally treated membranes. Figure S5. Gel contents of 6FDA-DCB: TAPA (3:2). Figure S6. Mechanical properties of 6FDA-DCB:TAPA(3:2) and 6FDA-DCB:TAPA(3:2)-450. (a) Stress-strain curves (b)Bending experiment. Table S1. Tensile strength and Elongation at break of 6FDA-DCB:TAPA(3:2) and 6FDA-DCB:TAPA(3:2)-450. Table S2. The pure gas diffusion coefficient (D), solubility coefficient (S), the diffusion selectivity (αS) and the solubility selectivity (αD) for all tested membranes at 0.2 MPa and 30 °C. Table S3. Pure gas permeabilities and ideal selectivities for 6FDA-DCB:TAPA(3:2)-450 and 6FDA-DABA:TAPA(3:2)-430 with different test pressures at 30 °C. Table S4. Physical aging performances of 6FDA-DCB:TAPA(3:2)-450 and 6FDA-DABA:TAPA(3:2)-430 aged 30, 60, 90, and 120 days at 0.2 MPa and 30 °C. Table S5. Mixed gas separation performance for 6FDA-DCB:TAPA(3:2)-450 and 6FDA-DABA:TAPA(3:2)-430 membranes at 0.2 MPa and 30 °C.
