# Supplementary material for: Tailoring the Micropore Structure of 6FDA-Based Network Polyimide Membranes for Advanced Gas Separation by Decarboxylation
Source: Membranes (Basel). 2023 Apr 24;13(5):461. doi: 10.3390/membranes13050461 (PMC10220741; doi:10.3390/membranes13050461)
Supplement: Supplementary file 1 [file membranes-13-00461-s001.zip › membranes-2300297-supplementary.pdf]

Supporting Information

Tailoring the micropore structure of 6FDA-based network polyimide membranes for advanced gas separation by decarboxylation

YuXuan Zhao, Hongyan Wang, Xiangyun Liu,*

Xueping Zong, Jiangzhou Luo ,Song Xue*

School of Chemistry & Chemical Engineering, Tianjin Key Laboratory of Organic Solar Cells and Photochemical Conversion, Tianjin University of Technology, Tianjin, 300384, China

*Corresponding authors: hywang@email.tjut.edu.cn; luojz@email.tjut.edu.cn

Table of Contents

1. Experimental Procedures

2. Supplementary Figures

3. Supplementary Tables

References

Section S1. Experimental Procedures

Section S1.1 Gel contents measurement

The gel contents of network PIs in DMF solvent were respectively measured by extracting the membrnaes with solvent for 24 h. The insoluble part was dried at 150 ºC for 24 h to constant weight. The gel contents were obtained via the following Equation

$$\text{Gel } \% = \frac{W_1}{W_0}$$

where W_0 and W_1 are the original weights and the insoluble part weights of the PI membranes, respectively [1-4].

Section S2. Supplementary Figures

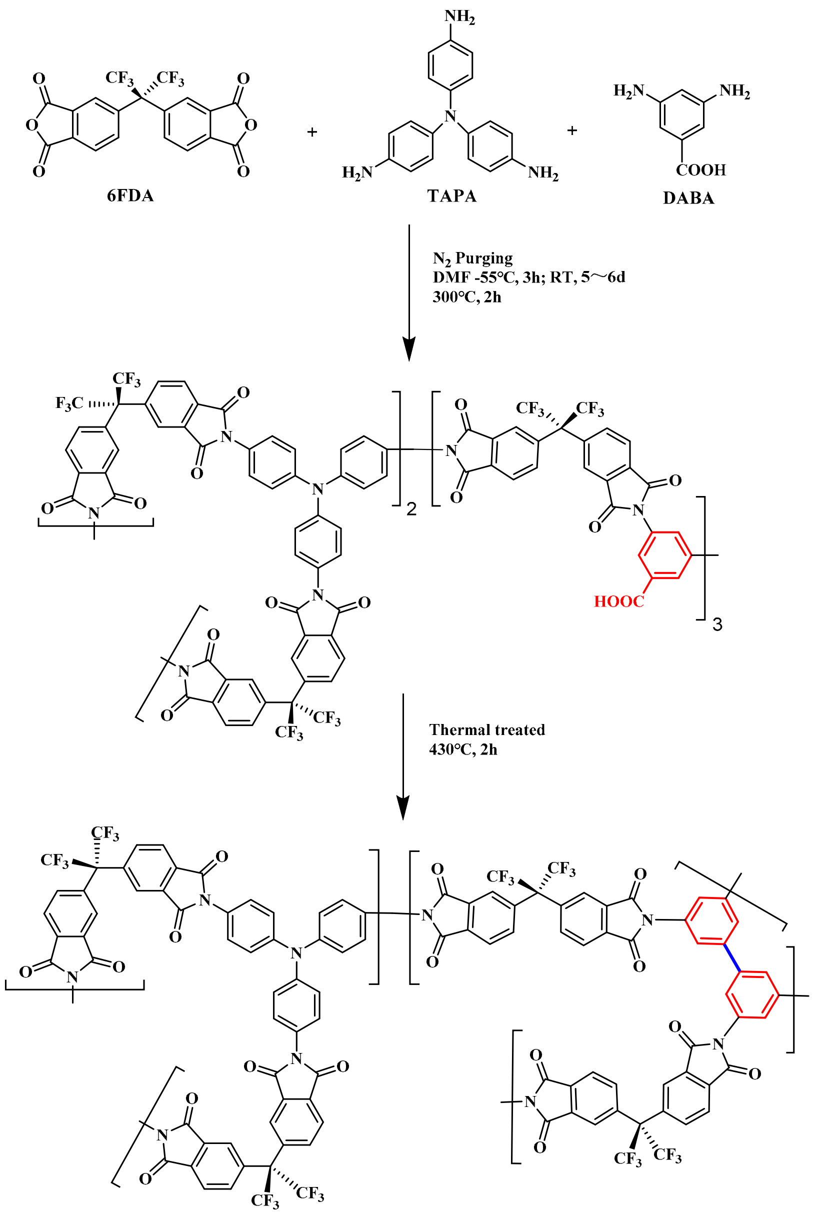

Scheme S1. Synthesis of 6FDA-DABA:TAPA(3:2) network PI and its thermally treated membrane 6FDA-DABA:TAPA(3:2)-430

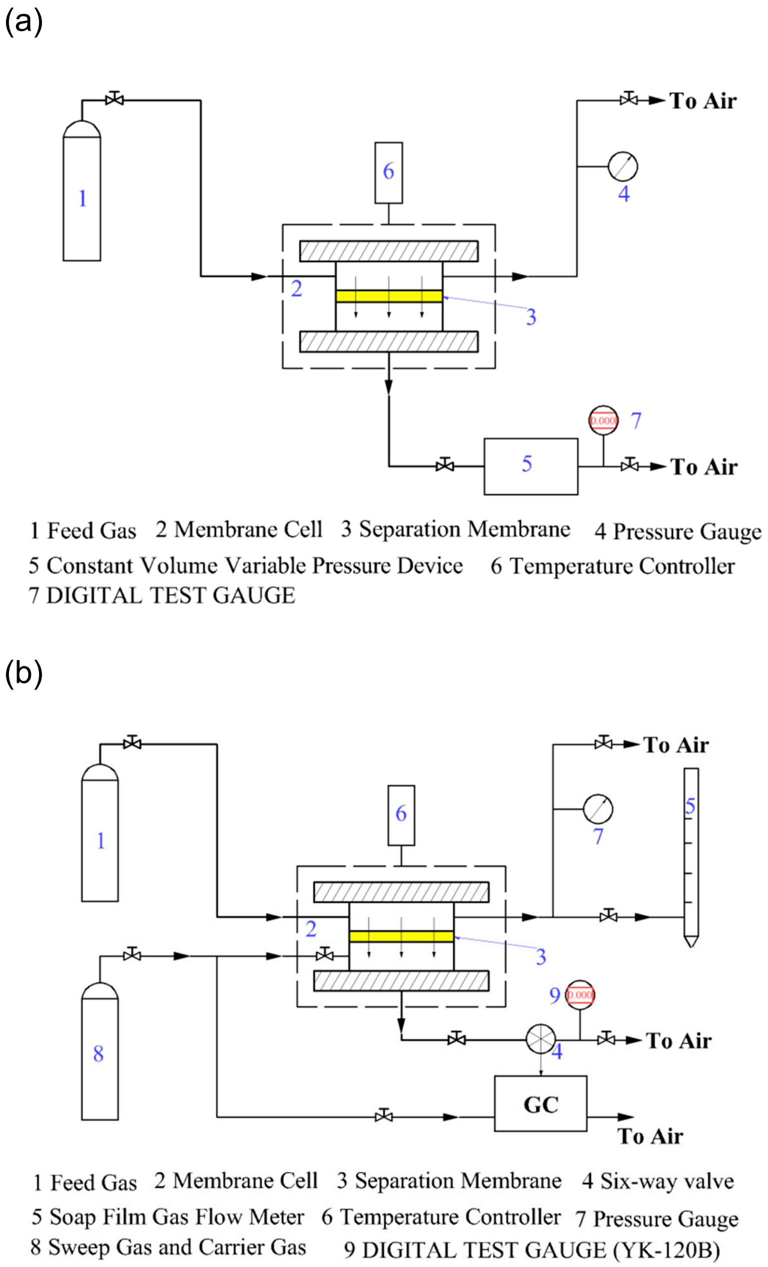

Figure S1. Flow chart of gas transport testing for **(a)** pure gas, **(b)** mixed gas.

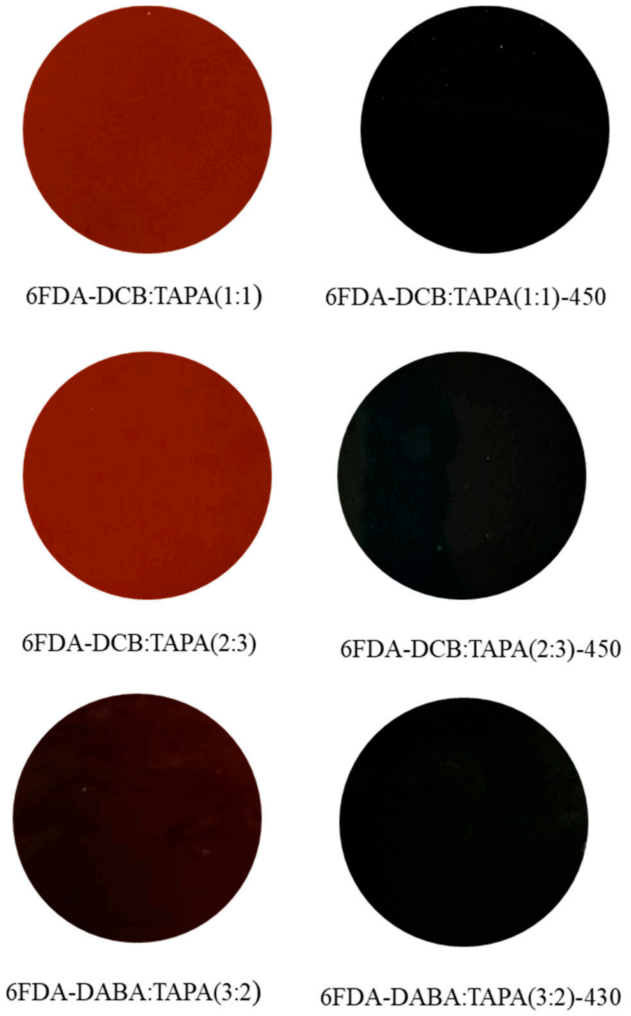

Figure S2. Optical pictures of prepared network PIs and their thermally treated membranes.

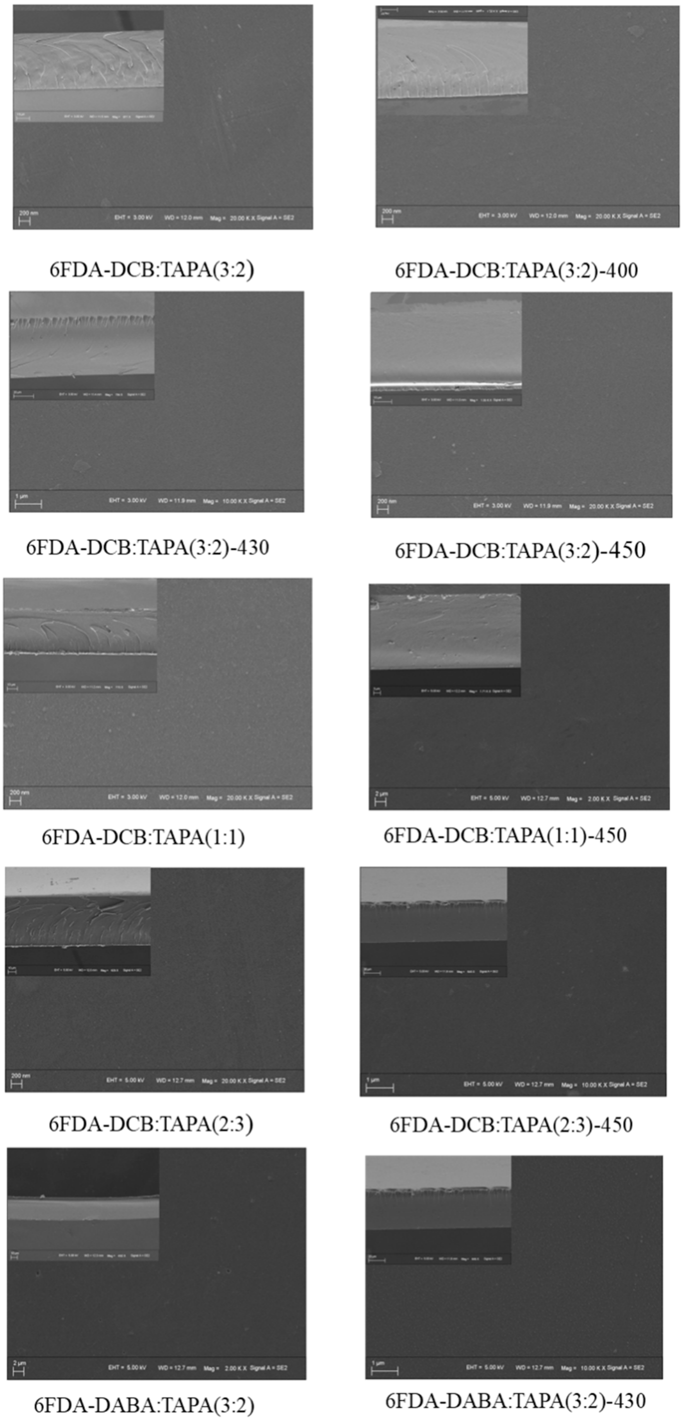

Figure S3. SEM images of prepared network PIs and their thermally treated membranes.

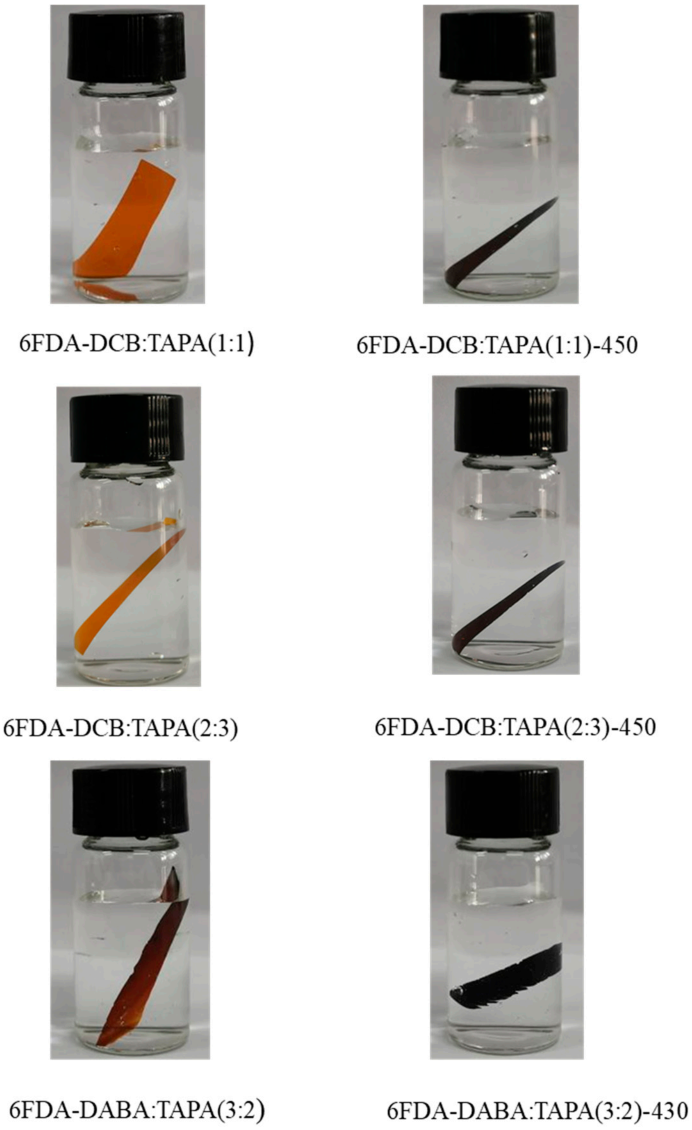

Figure S4. Solubility test of prepared network PIs and their thermally treated membranes.

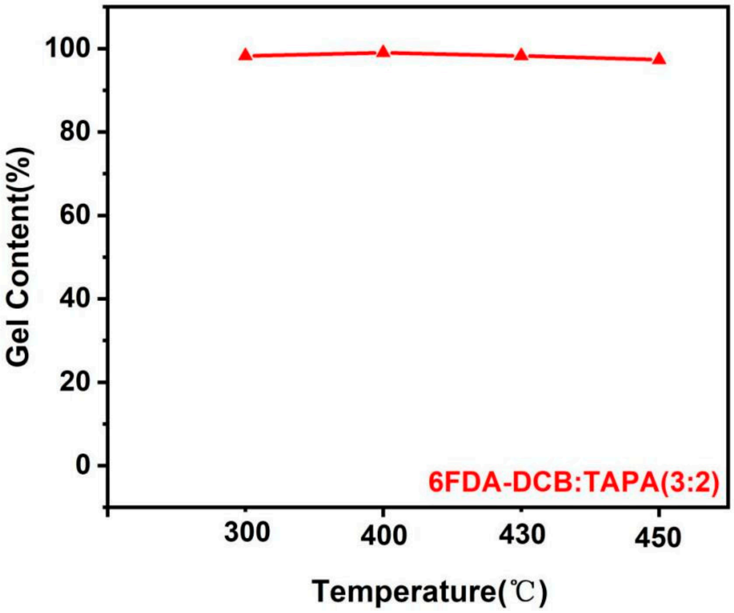

Figure S5. Gel contents of 6FDA-DCB: TAPA (3:2).

(a)

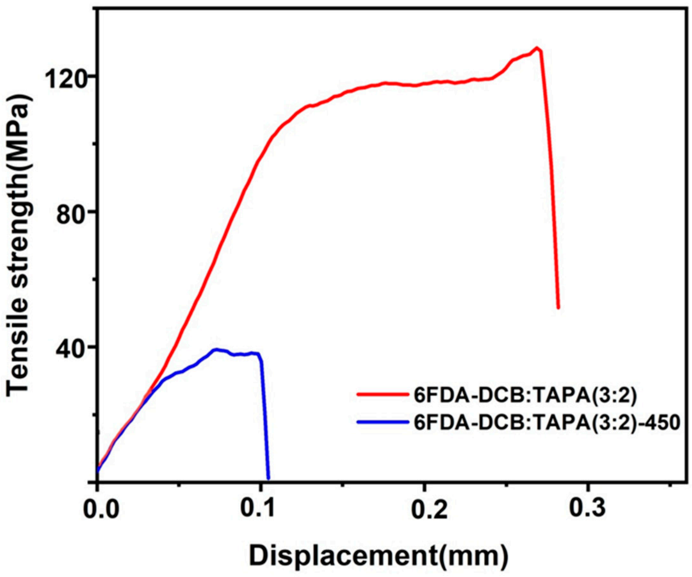

(b)

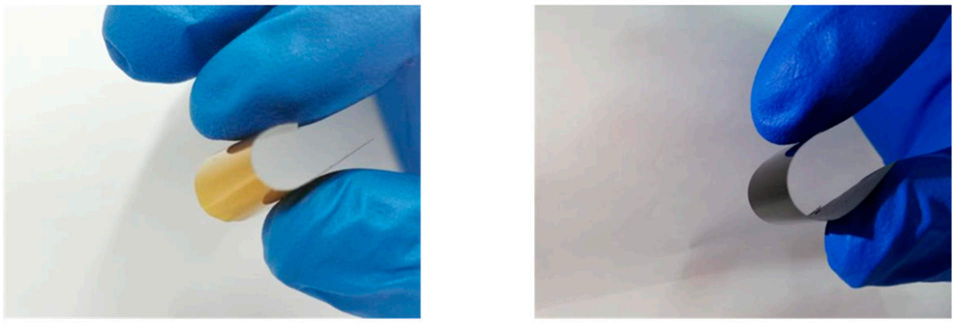

6FDA-DCB:TAPA(3:2) 6FDA-DCB:TAPA(3:2)-450

Figure S6. Mechanical properties of 6FDA-DCB:TAPA(3:2) and 6FDA-DCB:TAPA(3:2)-450. (a) Stress-strain curves (b)Bending experiment

Section S3. Supplementary Tables

Table S1. Tensile strength and Elongation at break of 6FDA-DCB:TAPA(3:2) and 6FDA-DCB:TAPA(3:2)-450.

PIs	Tensile strength(MPa)	Elongation at break(%)
6FDA-DCB:TAPA(3:2)	131	5.6
6FDA-DCB:TAPA(3:2)-450	38	2.2

Table S2. The pure gas diffusion coefficient (*D*), solubility coefficient (*S*), the diffusion selectivity (α_S) and the solubility selectivity (α_D) for all tested membranes at 0.2 MPa and 30 °C.

PIs	D ($10^{-8\cdot cm2\cdot s-1}$)				S ($10^{-2\cdot\ cm3}$) (STP)$\cdot cm^{-3}\cdot cmHg^{-1}$)			
	N_2	O_2	CO_2	α(CO_2/N_2)	N_2	O_2	CO_2	α(CO_2/N_2)
6FDA-DCB:TAPA(3:2)	1.1	3.15	7.2	6.5	0.96	2.79	5.44	5.6
6FDA-DCB:TAPA(3:2)-400	2.25	5.33	8.75	4	1.16	2.96	6.52	5.6
6FDA-DCB:TAPA(3:2)-430	3.23	6.48	12.43	3.9	1.32	3.27	7.1	5.4
6FDA-DCB:TAPA(3:2)-450	5.91	14.37	23.97	4.1	1.92	3.99	11.12	5.8
6FDA-DCB:TAPA(1:1)	1.1	2.98	6.71	6.1	1.44	3.1	6.93	4.8
6FDA-DCB:TAPA(1:1)-450	4.39	10.68	20.76	4.8	2.35	4.65	11.57	4.9
6FDA-DCB:TAPA(1:1)	1.22	3.23	7.14	6	1.41	3.16	6.83	4.8
6FDA-DCB:TAPA(1:1)-450	3.38	11.11	18.78	5.7	2.82	4.19	12.52	4.4
6FDA-DABA:TAPA(3:2)	1.19	2.6	6.43	5.8	1.17	3.91	5.32	4.5
6FDA-DABA:TAPA(3:2)-430	5.4	6.32	19.12	3.5	1.37	5.46	10.64	7.7

Table S3. Pure gas permeabilities and ideal selectivities for 6FDA-DCB:TAPA(3:2)-450 and 6FDA-DABA:TAPA(3:2)-430 with different test pressures at 30 °C.

PIs	Pressure (MPa)	P(Barrer)			α	
		**N_2**	**O_2**	**CO_2**	**O_2/N_2**	**CO_2/N_2**
6FDA-DCB:TAPA (3:2)-450	0.2	10.99	49.09	169.49	4.5	15.4
	0.4	10.07	46.3	135.64	4.6	13.5
	0.6	9.58	44.25	119.31	4.6	12.5
	0.8	8.74	40.09	98.55	4.6	10.3
	1.0	8.13	38.2	92.03	4.7	11.3
	1.2	7.83	37.58	87.3	4.8	11.1
6FDA-DABA:TAPA (3:2)-430	0.2	6.3	30.9	141.39	4.9	22.4
	0.4	5.97	26.54	115.9	4.4	19.4
	0.6	5.62	26.2	93.07	4.7	16.6
	0.8	5.23	25.66	86.03	4.9	16.4
	1.0	4.91	25.28	84.81	5.1	17.3
	1.2	4.85	25.02	78.57	5.2	16.2

1 Barrer= 10^{-10} (cm^3 (STP)·cm)/(cm^2·sec cmHg).

Table S4. Physical aging performances of 6FDA-DCB:TAPA(3:2)-450 and 6FDA-DABA:TAPA(3:2)-430 aged 30, 60, 90, and 120 days at 0.2 MPa and 30 °C.

PIs	Days	P(Barrer)			α	
		N_2	O_2	CO_2	O_2/N_2	CO_2/N_2
6FDA-DCB:TAPA (3:2)-450	0	11.32	57.29	266.6	5.1	23.6
	30	10.99	49.09	169.49	4.5	15.4
	60	9.4	43.87	138.14	4.7	14.7
	90	8.73	39.69	117.87	4.5	13.5
	120	8.29	37.41	102.35	4.5	12.3
6FDA-DABA:TAPA (3:2)-430	0	7.37	39.6	203.45	5.4	27.6
	30	6.3	30.9	141.39	4.9	22.4
	60	5.95	25.8	136.96	4.3	23
	90	5.51	23.71	130.67	4.3	23.7
	120	5.08	22.87	127.43	4.5	25.1

1 Barrer= 10^{-10} (cm^3 (STP)·cm)/(cm^2·sec cmHg).

Table S5. Mixed gas separation performance for 6FDA-DCB:TAPA(3:2)-450 and 6FDA-DABA:TAPA(3:2)-430 membranes at 0.2 MPa and 30 °C.

PIs	Mixed-gas CO_2/N_2	Permeate gas concentration (v/v)	P_{CO2} (Barrer)	P_{N2} (Barrer)	α
6FDA-DCB:TAPA (3:2)-450	15/85	80.8/19.2	230.66	9.67	23.9
6FDA-DABA:TAPA (3:2)-430	15/85	84.5/15.5	197.17	6.38	30.9

1 Barrer= 10^{-10} (cm^3 (STP)·cm)/(cm^2·sec cmHg).

Reference

[1] Y. Liu, R. Wang, T-S. Chung. Chemical cross-linking modification of polyimide membranes for gas separation, J. Membr. Sci. 189 (2001) 231-239.

[2] G.Q. Chen, C.A. Scholes, C.M. Doherty, A.J. Hill, G.G. Qiao, S.E. Kentish, The thickness dependence of Matrimid films in water vapor permeation, Chem. Eng. J. 209 (2012) 301–312, https://doi.org/10.1016/j.cej.2012.07.135.

[3] A. Bondi, Van der Waals Volumes and Radii, J. Phys. Chem. 68 (1964) 440-451, https://doi.org/10.1021/j100785a001.

[4] J.R. Wiegand, Z.P. Smith, Q. Liu, C.T. Patterson, B.D. Freeman, R. Guo, Synthesis and characterization of triptycene-based polyimides with tunable high fractional free volume for gas separation membranes, J. Mater. Chem. A. 2 (2014) 13309–13320, https://doi.org/10.1039/c4ta02303j.
